# Spatiotemporal Dynamics of Highly Pathogenic Avian Influenza Subtype H5N8 in Poultry Farms, South Korea

**DOI:** 10.3390/v13020274

**Published:** 2021-02-10

**Authors:** Woo-Hyun Kim, Sun Hak Bae, Seongbeom Cho

**Affiliations:** 1 College of Veterinary Medicine and Research Institute for Veterinary Science, Seoul National University, Seoul 08826, Korea; iceman2b@snu.ac.kr; 2 Department of Geography Education, Kangwon National University, Chuncheon 24341, Korea; gis119@kangwon.ac.kr

**Keywords:** avian influenza, H5N8 subtype, South Korea, spatiotemporal analysis

## Abstract

Highly pathogenic avian influenza (HPAI), a zoonotic disease, is a major threat to humans and poultry health worldwide. In January 2014, HPAI virus subtype H5N8 first infected poultry farms in South Korea, and 393 outbreaks, overall, were reported with enormous economic damage in the poultry industry. We analyzed the spatiotemporal distribution of HPAI H5N8 outbreaks in poultry farms using the global and local spatiotemporal interaction analyses in the first (January to July 2014) and second (September 2014 to June 2015) outbreak waves. The space–time K-function analyses revealed significant interactions within three days and in an over-40 km space–time window between the two study periods. The excess risk attributable value (*D*_0_) was maintained despite the distance in the case of HPAI H5N8 in South Korea. Eleven spatiotemporal clusters were identified, and the results showed that the HPAI introduction was from the southwestern region, and spread to the middle region, in South Korea. This spatiotemporal interaction indicates that the HPAI epidemic in South Korea was mostly characterized by short period transmission, regardless of the distance. This finding supports strict control strategies such as preemptive depopulation, and poultry movement tracking. Further studies are needed to understand HPAI disease transmission patterns.

## 1. Introduction

Highly pathogenic avian influenza (HPAI) is a major zoonotic disease that threatens public health [1]. The HPAI virus (HPAIV) is highly contagious to domestic poultry and continuously occurs worldwide, causing enormous damage in the poultry industry [2]. The HPAI subtype H5N8 infection in poultry farms was first reported in January 2014 in South Korea [3]. The results of a genetic epidemiologic investigation showed that the infection transmission occurs through the migratory pathway of wild birds in the winter season, indicating that the introduction of H5N8 HPAIV is associated with the wild waterbirds [4]. Migratory birds that stay in South Korea move through the East Asia–Australia flyway, and the HPAI H5N8 virus has disseminated to other continents, including Europe and the United States, through the overlying flyways of migratory birds [5].

While the wild migratory birds are the source of viral infection in domestic poultry farms [6], HPAIV transmission and spread between farms occurs mechanically through transport vehicles, people, feeds, clothes, shoes, and equipment, contaminated by dust, water, and feces of HPAIV-infected poultry [7]. In a recent study, HPAIV airborne transmission was considered possible between poultry farms and may have played a role in the spread of HPAI outbreaks in the United States [8]. Considering these various HPAIV transmission pathways, it is important to understand how HPAI disease is transmitted through time and space, to understand and prevent the spread of disease.

The occurrence of space–time interactions between outbreak cases located close in time and space varies and can be considered an infectious disease indicator [9]. Measuring and analyzing this indicator provides an understanding of the disease’s underlying mechanism, which enables the development of prevention strategies against disease spread [10]. Space–time interaction analysis using the space–time K function has been used in foot-and-mouth disease in the United Kingdom [11,12] and Tanzania [13], bovine tuberculosis in New Zealand [14], Rift Valley fever in South Africa [15], and Africa swine fever in Russia Federation [16]. The spatiotemporal interaction of HPAI has been studied in France for the H5N8 subtype [17] and in Vietnam for the H5N1 subtype [18].

This study aimed to identify the time and space distribution of HPAI H5N8 outbreaks in South Korea from 2014 to 2016. The space–time interaction was analyzed using the space–time K function analysis and the scan statistics of HPAI transmission dynamics. It is believed that this systematic understanding of the spatiotemporal distribution will enable the evaluation of quarantine policies to address the HPAI outbreaks, thereby providing scientific evidence for future policy development and suggestion of the direction for further research.

## 2. Materials and Methods

### 2.1. Data Collection and Management

The epidemic data of HPAI subtype H5N8 were collected by the Animal and Plant Quarantine Agency (APQA) in Gimcheon, South Korea, from 15 January 2014 to 5 April 2016 [19]. Following the identification of birds with clinical signs suspicious of HPAI infection by the livestock owners, farmworkers, and veterinarians, the case must be reported to the APQA according to the Act on Prevention of Contagious Animal Diseases [20] in passive surveillance. Veterinarians from governmental agencies visited the reported poultry farms to collect samples from the sick or dead birds, and then samples were tested to confirm possible HPAI infection. If the suspected farm was confirmed as HPAI-positive, it was deemed an infected premise (IP). Then infected poultry farms and neighboring farms located within a protective zone set to a radius of 3 km were depopulated. A depopulated farm found to be positive for HPAIV was referred to as a positive premise (PP) [21] in active surveillance.

In Korea, all the transporting vehicles related to the poultry industry for transporting either poultry, poultry products, medicines, feed, or feces must be registered with the Korea Animal Health Integrated System (KAHIS; http://www.kahis.go.kr, accessed on 9 September 2020). Based on these vehicles’ geographical information, APQA conducted an HPAI diagnostic test on the poultry farms visited by the vehicles entering HPAI-infected farms. This active epidemiological investigation of livestock-related vehicle movement makes it possible to detect additional HPAI-infected farms. In this study, all IPs and PPs found through this surveillance were considered as cases. All the geographical data of the poultry farms that were collected at the Tong-ri Administration, village levels, were projected to WGS84/UTM zone 52N (European Petroleum Survey Group; EPSG: 32652) and processed using QGIS 3.4 [22]. The date for each case was the date the first clinical signs were observed. If no clinical signs indicative of HPAI were observed in the farms’ poultry, the date of cases was based on the day of HPAI positive confirmation.

### 2.2. Spatiotemporal Analysis

HPAI subtype H5N8 outbreak from 15 January 2014 to 5 April 2016 was classified into four waves in Korea [19]. In this study, the first wave from 15 January 2014 to 29 July 2014 and the second wave from 24 September 2014 to 10 June 2015, were analyzed. The third (17 cases) and fourth waves (2 cases) of H5N8 were excluded from the analysis due to the inadequate number of cases.

Global and local spatiotemporal interaction analyses were conducted to describe the differences in spatiotemporal characteristics of HPAI subtype H5N8 between poultry farms in Korea. The global analysis used the space–time K function to calculate the spatiotemporal interactions of HPAI H5N8 outbreaks [9]. The space–time K function, K(s,t), was defined as the number of expected cases (E), if cases are randomly-distributed within a distance s and a time t, then divided by the intensity λ, defined as the mean number of cases per unit of space and time (Equation (1)).

K(s,t) = λ^(−1) E(1)

If cases occur independently in time and space without a space–time interaction, K(s,t) was the product of two K functions in space and time, similar to that shown in Equation (2).

K(s,t) = K(s)*K(t)(2)

We defined D(s,t) as the difference between the observed and randomly expected space–time interactions (Equation (3));

D(s,t) = K(s,t) − K(s)*K(t).(3)

In this Equation (3), D(s,t) > 0 means that space–time interactions are presented at a distance s and time t; with higher D(s,t) values showing stronger evidence. *D*_0_ is the value interpreted as the proportional increase, or excess risk attributable to the space–time interaction to facilitate inference (Equation (4)).

*D*_0_(s,t) = D(s,t)/(K(s)*K(t)) (4)

*D*_0_(s,t) > 1 indicates that the number of observed events is greater than twice the number of expected events [9,11].

The null hypothesis of no space–time interaction in the observed cases was tested, the dates of the cases were randomly permuted on a fixed set of the location of the cases, using Monte-Carlo simulation, to generate a distribution of D(s,t), to compare with the D(s,t) of the observed cases.

Suppose D(s,t) values for observed cases exceeds 95% of the values derived from the simulation; then, we reject the null hypothesis because the probability of observed space–time interaction occurring by chance is less than the 5% probability. Therefore, it can then be concluded that there was a significant space–time interaction between the observed cases.

In this study, global spatiotemporal clustering of HPAI H5N8 outbreaks was investigated in the first and second study periods using the space–time K function [15]. The space–time K function analysis was conducted using the maximum space–time window of 40 km and 40 days. Significant space–time clustering was simulated by generating 999 Monte-Carlo random permutations. The *D*_0_(s,t) value, the excess risk attributable to the space–time interaction within a distance s and time t, was calculated and visualized in R software version 3.6 (R Project for Statistical Computing, Vienna, Austria) [23] using the “splancs” package [24].

We used the space–time permutation model of the scan statistics to identify the local spatiotemporal cluster of HPAI H5N8 outbreaks [25], by applying the spatiotemporal windows shown in the global spatiotemporal clustering. This approach was performed by creating a series of hypothetical spatiotemporal cylinder-centered coordinates for each case [26]. These cylinder bases and heights represent the space and time dimensions of each potential cluster, respectively. To calculate the cylinder, the approach used was to a finite number iteration and then, a gradual increase in the circle radius and height from zero to the maximum space and time value defined by the user. To test the null hypothesis, which assumed a no space–time interaction between cases, randomly distributed permutation of the spatial and temporal attributes of each case were performed using the Monte-Carlo simulation. Through this simulation, the expected disease occurrence was obtained when time and space were assumed to be independent of each other within a given space and time frame. If the observed number of the actual cases are higher than the expected number of cases calculated through the above process, it is then inferred that the number of cases in the region within the cluster is more frequent in space and time than the rest of the cases in the geographic areas [13,14,26].

The presence of local spatiotemporal clusters in HPAI H5N8 during the two study periods (first and second waves of the outbreaks) between the case poultry farms was investigated using the space–time permutation model of the scan statistic test, implemented using the SatScan (New York, NY, USA) [26]. Statistically significant difference was reported at the 5% level, assessed by the 999 Monte-Carlo replications without overlapping. The maximum spatiotemporal window was set to 25% of the number of outbreak cases (first wave, 53 cases; second wave, 41 cases) and 25% of the study period (first wave, 49 days; second wave, 75 days).

## 3. Results

### 3.1. Descriptive Analysis

In total, 393 HPAI subtype H5N8 outbreaks were reported in poultry farms from 15 January 2014 to 5 April 2016. Of these outbreaks, the first wave occurred from 15 January 2014 to 29 July 2014 while the second wave was from 24 September 2014 to 10 June 2015. During these two study periods, most outbreaks occurred among ducks (75.7%, 283/374), followed by chickens (20.9%, 78/374), and then others (3.5%, 13/374) such as quail or ostriches. In the first (78.3%) and second waves (72.2%), the outbreaks mainly infected ducks (Table 1).

**Figure 1:**
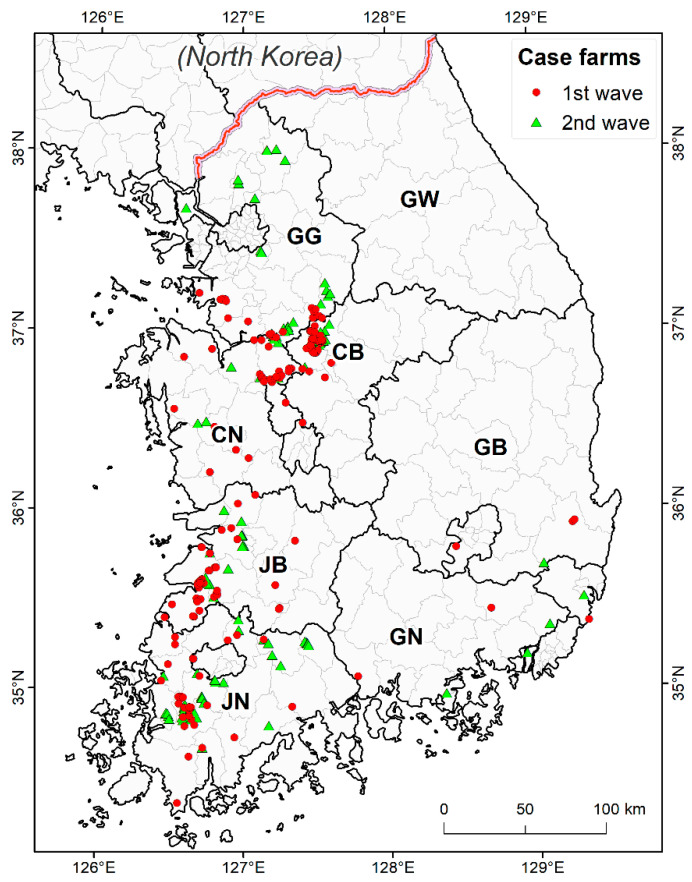
Location of highly pathogenic avian influenza case farms in the first and second waves. Red round dots are the outbreaks in poultry farms during the first wave from 14 January 2014 to 29 July 2014. Green triangles are the outbreaks in poultry farms in the second wave from 23 September 2014 to 24 June 2015. Province abbreviations: CB: Chungbuk, CN: Chungnam, GB: Gyeongbuk, GG: Gyeonggi, GN: Gyeongnam, GW: Gangwon, JB: Jeonbuk, JN: Jeonnam.

HPAI H5N8 was distributed nationwide but was mainly concentrated in the west coastal and southern regions where the domestic duck breeding density was high [27] (Figure 1). The order of the intensities of the distribution by province were Jeollanam-do (JN) (28.6%, 107/374), Chungcheongbuk-do (CB) (24.9%, 93/374), and Jeollabuk-do (JB) (19.0%, 71/374). The order of the case incidence rates was CB (27.4%), JB (22.2%), and JN (22.2%) in the first H5N8 outbreak period; and JN (37.0%), CB (21.6%), and Gyeonggi-do (GG) (19.1%), in the second outbreak period.

**Figure 2:**
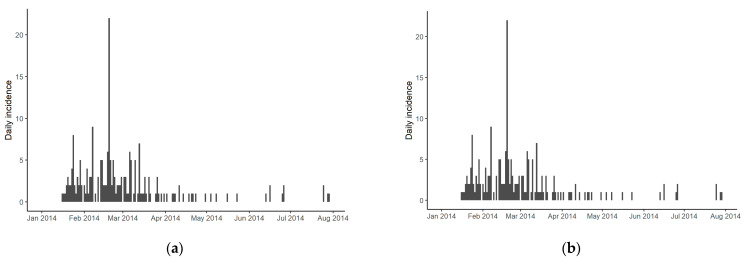
Epidemic curve of HPAI H5N8 from 2014–2015: (**a**) the epidemic curve of the first wave of HPAI H5N8 is from January 2014 to August 2014 in South Korea; (**b**) the epidemic curve of the second wave of HPAI H5N8 is from September 2014 to July 2015 in South Korea. HPAI, highly pathogenic avian influenza.

The temporal distributions of the first and second outbreaks of HPAI H5N8 in Korea are shown in Figure 2. After the first case farm outbreak was reported on 16 January 2014, the outbreaks increased continuously, peaked in February 2014, and only intermittently spread after 20 March 2014 (Figure 2a). The number of poultry farms infected during the exponential period was 178 (of 212 cases, 83.9%) in the first study period. In the second study period, the HPAIV was reintroduced to Korea on 24 September 2014, with a total of 61 farm outbreaks (37.6%, 61/162), which were infected exponentially for 34 days from 28 January 2015 to 3 March 2015 (Figure 2b).

### 3.2. Spatiotemporal Analysis

**Figure 3:**
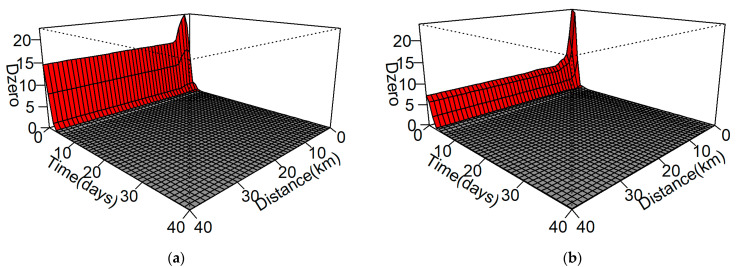
Excess risk attributable to the space–time interaction (*D*_0_) as a function of space and time. (**a**) Excess risk attributable to the space–time interaction of the first wave of HPAI H5N8 from 14 January 2014 to 29 July 2014. (**b**) Excess risk attributable to space–time interaction of the second wave of HPAI H5N8 from 23 September 2014 to 24 June 2015. The red-shaded area shows the space–time interaction for which the observed number of cases was higher than twice the expected number, which assumes no space–time interaction (*D*_0_ > 1). HPAI, highly pathogenic avian influenza.

Out of the 393 outbreaks of HPAI H5N8, a spatiotemporal analysis was performed on 212 and 162 farm outbreaks during the first and second study periods, respectively. The global spatiotemporal cluster of HPAI H5N8 in poultry farms was statistically significant (*p* < 0.05) for each study period (Figure 3). During the first study period (15 January 2014–29 July 2014), the excess risk attributable to space–time interaction with *D*_0_ > 1 was a 40-km space–time window and 3 days; the time was closer to 0, and the *D*_0_ value was higher (Figure 3a). The *D*_0_ value was the highest (21.4) at the spatiotemporal parameters of a 2-km space–time window and 0 days, and when the temporal parameter was set as 0 days, the *D*_0_ value was maintained at 15, despite the increasing distance.

The excess risk attributable to the spatiotemporal interaction in the second study period (24 September 2014–10 June 2015) was a 40 km space–time window and 3 days; the time was closer to 0, and the *D*_0_ value was higher (Figure 3b). The excess risk attributable during the second period had a similar pattern to that of the first study period. The *D*_0_ value was the highest (23.4) in the 2 km space–time window and 0 days, and the value of 6 was maintained, despite the increasing distance.

**Figure 4:**
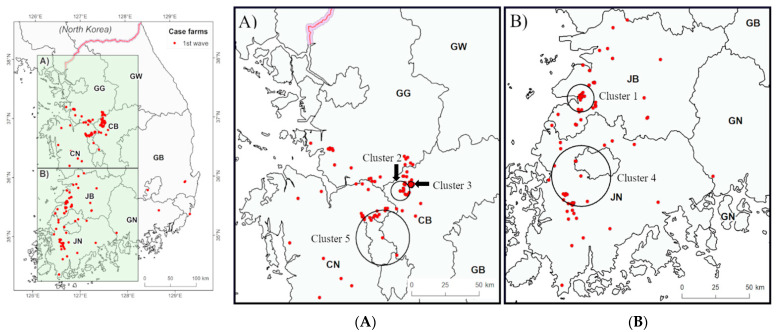
Spatiotemporal cluster of poultry farms during the first wave of HPAI H5N8 outbreaks in Korea. The left map shows whole area of South Korea; (**A**) spatiotemporal cluster of the first wave of HPAI H5N8 in the midwest region; (**B**) spatiotemporal cluster of the first wave of HPAI H5N8 in the southwest region. Red round dots are the outbreaks in poultry farms at the first wave from 14 January 2014 to 29 July 2014. The locations of the clusters are indicated by black circles and clusters name is shown by black arrow. Province abbreviations: CB: Chungbuk, CN: Chungnam, GB: Gyeongbuk, GG: Gyeonggi, GN: Gyeongnam, GW: Gangwon, JB: Jeonbuk, JN: Jeonnam.

**Figure 5:**
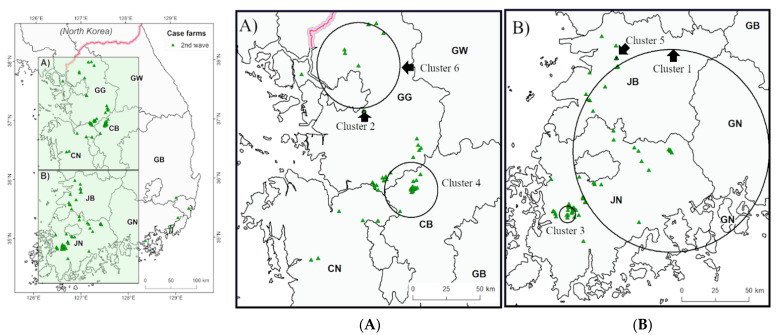
Spatiotemporal cluster of poultry farms during the second wave of HPAI H5N8 outbreaks in Korea. The left map shows whole area of South Korea; (**A**) spatiotemporal cluster of the second wave of HPAI H5N8 in the midwest region; (**B**) spatiotemporal cluster of the second wave of HPAI H5N8 in the southwest region. Green triangles are the outbreaks in poultry farms at the second wave from 23 September 2014 to 24 June 2015. The locations of the clusters are indicated by black circles and clusters name is shown by black arrow. Province abbreviations: CB: Chungbuk, CN: Chungnam, GB: Gyeongbuk, GG: Gyeonggi, GN: Gyeongnam, GW: Gangwon, JB: Jeonbuk, JN: Jeonnam.

We identified the 11 statistically significant spatiotemporal clusters from the result of the space–time permutation scan statistic test. The geographical location of each cluster, numbered according to the time of occurrence, is indicated in Figure 4 and Figure 5. The radius (km), temporal extension (days), number of outbreaks in the cluster, and the observed to the expected ratio of each cluster are shown in Table 2. The clusters were mainly formed around the west coastal area in South Korea. In the first study period, two clusters (Figure 4A) were formed in JN and JB, while three clusters were formed around the border areas of GG, CB, and CN (Figure 4B). The maximum spatial expansion of the clusters ranged from 2.21 to 24.84 km, and the maximum time ranged from 3 to 30 days (Table 2). The Cluster 3 was the smallest among the clusters in the first period (2.21 km), but the number of farms included in this cluster was the largest (28 cases). In the second study period, three clusters were found in JN and JB (Figure 5A), two clusters in the northern GG regions, and one cluster in the border areas of CB, CN, and GG (Figure 5B). The maximum space of the cluster was between 0.46 and 72.59 km, while the duration was between 5 and 36 days (Table 2). All the clusters that showed statistically significant difference during the first study period overlapped with an epidemic exponential growth period (16 January–20 March 2014), while only one statistically significant cluster was found to have overlapped with the second exponential period (28 January–3 March 2015). Among HPAI poultry farms, during the study period, the proportion of farms in the cluster that were derived from the scan statistic test was 48.11% (102/212) in the first, and 51.23% (83/162) in the second.

## 4. Discussion

It is important to identify and analyze clustering to detect the area with a higher level of disease risk during outbreak investigations [28]. There have been many attempts to apply spatiotemporal modelling to zoonosis, by estimating the space–time interaction between cases that are spatially and temporally proximate, making it possible to interpret the underlying transmission process [13,17,29]. Despite the importance of understanding the spatiotemporal disease dynamics, epidemiological research into HPAI epidemics in Korea was mainly focused on molecular investigations to track the origin of HPAIV strains and pathogens [30,31]. It is important to analyze the global and local spatiotemporal interaction for the HPAI H5N8 outbreak to understand the disease transmission process for effective HPAI controls in poultry farms. This study investigated the spatiotemporal patterns of the first and second waves that occurred after the H5N8 HPAIV was introduced to Korea in January 2014. As far as we know, this is the first HPAI subtype H5N8 study in Korea that analyzed the global and local space–time interaction. This result will be a cornerstone in explaining the spatiotemporal factors related to HPAI H5N8 infection and transmission.

In space–time K function analysis, we identified space–time interactions over a distance of more than 40 km and under two days in the first study period (Figure 3A). In addition, at 2 km and 0 days, the risk was highest and then decreased, maintaining a constant risk regardless of the increasing distance. This pattern of the space–time interaction was the same in the second study period, but the peak of the risk was highest at 0 days and 0 km, and the *D*_0_ value decreased from 15 to 6 after two days (Figure 3B). These results showed a different pattern from those of previous research [17,18], which performed the spatiotemporal analyses for other HPAIs, and the pattern seems to be characteristic of HPAI disease transmission in Korea. The results of the space–time K function analysis for HPAI in other countries showed significant spatiotemporal clustering less than 13 days and 8 km in France [17], and less than 50 days and 60 km in Vietnam [18]. Moreover, the excess risk reported in both studies showed a pyramidal shape, in which the *D*_0_ decreased as time and distance increased. Conversely, the excess risk was maintained even when the distance was increased in the spatiotemporal interaction in Korea (Figure 3). These results imply that the spread of HPAI disease in Korea occurs consistently regardless of the distance, especially within two days.

According to the results of our local spatiotemporal cluster analysis, HPAI from our two study periods tends to appear in clusters in the western coastal area in Korea (Figure 4 and Figure 5). Five spatiotemporal clusters were shown from 0.46 to 24.84 km and from 5 to 28 days in JN and JB Provinces (Figure 4B and Figure 5B). Among them, cluster 1 appeared in the early stages of each of the outbreak waves, and in the southwestern coastal area, the major habitats of the wild migratory birds in the winter season. After introduction or re-introduction of HPAIV into Korea, six clusters from 2.21 to 72.59 km and from 3 to 36 days were formed in the three provinces of GG, CB, and CN (Figure 4A and Figure 5A). The assumption is that the HPAI introduced from the southwest region spread to the central area, considering the cluster formation time. The results of the spatiotemporal clustering of HPAI H5N8 are consistent with the results of the investigation of the origin and transmission of H5N8 by sequencing analysis, indicating that H5N8 virus entered into the western coastal provinces and spread rapidly to other provinces with high densities of winter migratory birds and ducks holding [27]. Considering these results, an intensive HPAI monitoring is necessary for these regions in winter seasons.

In our results, six clusters were distributed from 0.46 to 9.86 km in space and from 3 to 19 days in time, while five clusters were distributed from 19.74 to 72.59 km in space and from 27 to 36 days in time (Table 2). Furthermore, cluster 3 in the first study period and cluster 3 and 5 in the second study period were less than 3 km in size. It might be that this phenomenon appeared as the size of clusters was suppressed by preemptive depopulation. On the contrary, 5 of the 11 clusters were covered the spatiotemporal extension from 27 to 36 days and from 19.74 to 72.59 km, which is greater than the period (10–25 days) and the distance (16.5 km–52.7 km) in the previous study on H5N8 spatiotemporal cluster analysis in France [17]. This shows that the regional spread of H5N8 in Korea was polarized between small and large spatiotemporal clusters. In other words, the HPAIV was disseminated over an extremely short distance and time, or rather spread over long-distances and times.

Based on the results of the global and local spatiotemporal interaction, the following were assumed to have affected the spatial and temporal characteristics of the HPAI H5N8 in the poultry farms. First, we assumed that HPAI reporting and depopulation are carried out quickly in Korea, which leads to the prevention of adjacent disease spread (by the preemptive depopulation), from infected farms to the neighboring poultry farms. This can be inferred from the results showing that the time window of excess risk from the time–space interaction analysis was two days, which is shorter than the time reported in other studies [17,18]. In addition, the third cluster in the first period, and the third and fifth clusters in the second period, had small spatial windows less than 3 km. If the HPAI report from the poultry farms and the disease quarantine were delayed, HPAI would have had sufficient opportunity to propagate adjacent poultry holdings, which would have shown a similar spatiotemporal interaction as those of other studies [17,18]. According to APQA, when the suspected poultry with clinical signs is reported and confirmed as positive, a 3-km radius depopulation is carried out around infected holdings, and this process, from report to depopulation, is conducted within a short period [19]. It can be inferred that the virus short-range contiguous transmission was blocked effectively by removing the host that could cause increased infection spread.

Second, considering the constant *D*_0_ regardless of distance, there is a high possibility that the cases that were due to the long-distance propagation of HPAI through vehicles were relatively due to oversampling, because of the suppression of the adjacent propagation of HPAI. The KAHIS was established in 2013 to integrate the management of animal disease and livestock quarantine information using information and communication technology (ICT) to prevent livestock disease outbreak [20]. It is possible to collect information on registered vehicle movements related to the poultry industry, such as feces treatment, veterinarian visits, and the transporting of feed, medicine, poultry, and poultry products. It is mandatory to a equip global positioning system (GPS) on registered vehicles under the Korean Act on the Prevention of Contagious Animal Diseases, and their movement information is periodically collected through KAHIS [32]. This systemic tracking makes it possible to track the HPAI long-distance dissemination. If an HPAI case found through the long-distance propagation tracking using this mechanical relationship is included in the analysis, it is judged that a pattern of the *D*_0_ value that is not affected by the distance in the spatiotemporal interaction can appear as shown in Figure 3. Indeed, epidemiological investigations conducted by the APQA have confirmed that the long-range propagation of the HPAI subtype H5N8 is largely due to the movement of the livestock vehicles. According to the results of the epidemiological investigations, 76.27% (299/392) of poultry farm infection was caused by the vehicles [19]. Therefore, this result of the global spatiotemporal interaction is presumed to rapidly suppress HPAI outbreaks through active surveillance.

Finally, the excess risk *D*_0_ in the second study period was relatively reduced compared to the *D*_0_ in the first study period (Figure 3). The factors that are estimated to have influenced the decrease in *D*_0_ during the second study period are as follows. First, the livestock owners may be already aware of the HPAI introduction in poultry holdings during HPAI recurrence. Through this recognition, it can be assumed that alertness to HPAI has increased and faster disease reporting has been carried out. The effect of knowledge and awareness to HPAI reporting was reported from a study of HPAI during the 2006–2008 outbreaks in Nigeria [33]. Second, the changed quarantine policy of the Korean government in the second period might be more effective in controlling the outbreak than in the first period. Due to continuous HPAI outbreaks in Korea, the Disease Outbreak Law and infectious disease standard operating procedure was revised in 2015 [20] and the systematic investigation of diseases was developed through the manual [34]. This change in HPAI biosecurity policy may have resulted in a reduction in excess risk attributable.

The finding of this study may have been affected by several limitations. First, there may have been cases where the presence of HPAI H5N8 was not reported if the sensitivity of the reports from the farms to the government were not optimal. In particular, in case of HPAI H5N8 in Korea, the infected ducks did not show clinical signs, which may have made the detection of H5N8 challenging [31,35]. However, it is already mandatory to sample the poultry at the farm a day before transportation to other farms or slaughterhouses, to inspect them for HPAI using reverse transcription polymerase chain reaction (RT-PCR), since 2008 [20]. Therefore, considering the massive HPAI inspection, the risk of unreported cases may be considered relatively low.

Second, our analysis was performed on the assumption that the date when the clinical signs were first observed was the date HPAIV was first introduced into the poultry farm. This may have had effects on the study results because the incubation period of HPAI H5N8 may differ depending on the poultry specie or the condition of the flocks. However, during our study periods, 75.7% of the cases included ducks; therefore, it can be assumed that the latent period of H5N8 will be similar in most of the poultry farms. The interval from the date of the virus introduction to the flocks to the date when the first clinical signs were observed is likely to be constant. Therefore, our assumption would not have had a significant impact on the temporal elements in our spatiotemporal analyses to the extent of results bias.

## 5. Conclusions

This study provides insights into the 2014–2016 Korea HPAI epidemic dynamics. This global and local spatiotemporal interaction indicates that the HPAI H5N8 epidemic in Korea was mostly characterized by short period dispersion within two days and occurred consistently regardless of the distance. This disease transmission pattern is different from other HPAI spatiotemporal interaction studies. It is believed that these spatiotemporal analysis results are closely related to the rapid preemptive depopulation, standstill, and disease tracking policy, using GPS. This finding supports the need for strict control strategies such as the preemptive depopulation, the standstill of poultry transporting, and the epidemiological movement tracking in Korea during the H5N8 disease period. Further research is needed to evaluate the optimal culling radius, the effects of wild birds in HPAI outbreaks, the spread rate of disease between farms, and the disease transmission pathways by poultry-related vehicles, to help understand HPAI disease transmission patterns.

## Figures and Tables

**Table 1:** Distribution of HPAI H5N8 outbreaks per specie, type, and period in Korea.

Wave	Duck (%)	Chicken (%)	Others (%)	Total Number of Cases	Starts	End	Number of Days
First	166 (78.3)	39 (18.4)	7 (3.3)	212	15 January 2014	29 July 2014	196
Second	117 (72.2)	39 (24.1)	6 (3.7)	162	24 September 2014	10 June 2015	260
Third	14 (82.4)	0 (0.0)	3 (17.6)	17	14 September 2015	15 November 2015	63
Fourth	2 (100.0)	0 (0.0)	0 (0.0)	2	23 March 2016	05 April 2016	14
Total	299 (76.1)	78 (19.8)	16 (4.1)	393			533

HPAI, highly pathogenic avian influenza.

**Table 2:** Spatiotemporal cluster of Korea poultry farms during HPAI H5N8 outbreaks from 2014 to 2016.

Wave	Cluster	Radius (km)	Start	End	Number of Days	Number of Outbreaks	Expected Outbreaks	Observed to Expected Ratio	*p*-Value
First	1	9.86	15 January 2014	24 January 2014	10	20	2.3	8.8	0.001
	2	6.91	01 February 2014	07 February 2014	7	15	1.8	8.5	0.001
	3	2.21	17 February 2014	19 February 2014	3	28	4.1	6.8	0.001
	4	24.84	10 March 2014	06 April 2014	28	17	3.9	4.4	0.002
	5	19.74	10 March 2014	08 April 2014	30	11	2.2	5.1	0.030
Second	1	5.95	24 September 2014	12 October 2014	19	14	1.9	7.2	0.001
	2	72.59	17 October 2014	19 November 2014	34	11	2.3	4.8	0.025
	3	1.30	22 December 2014	26 December 2014	5	5	0.2	32.5	0.001
	4	19.76	22 February 2015	20 March 2015	27	36	10.2	3.5	0.001
	5	0.46	26 March 2015	12 April 2015	18	11	0.8	13.5	0.001
	6	30.74	16 April 2015	21 May 2015	36	6	0.4	16.2	0.001

HPAI, highly pathogenic avian influenza.

## Data Availability

Publicly available datasets were analyzed in this study. This data can be found here: https://ebook.qia.go.kr/home/view.php?host=main&site=20161018_132952&listPageNow=0&list2PageNow=0&code=0&code2=0&code3=0&optionlisttype=&searchcode=0&searchcode2=0&searchdate=0&searchkey=all&searchval=%C1%B6%B7%F9%C0%CE%C7%C3%B7%E7%BF%A3%C0%DA&searchandor=&dummy=&&orders= (accessed on 9 September 2020).
